# Devastating intimacy: the cell biology of plant–*Phytophthora* interactions

**DOI:** 10.1111/nph.16650

**Published:** 2020-06-19

**Authors:** Petra C. Boevink, Paul R. J. Birch, Dionne Turnbull, Stephen C. Whisson

**Affiliations:** ^1^ Cell and Molecular Sciences James Hutton Institute Errol Road Invergowrie Dundee DD2 5DA UK; ^2^ Division of Plant Sciences University of Dundee Errol Road Invergowrie Dundee DD2 5DA UK

**Keywords:** effector, haustoria, *Phytophthora*, plant defence, plant–pathogen interactions, RXLR

## Abstract

An understanding of the cell biology underlying the burgeoning molecular genetic and genomic knowledge of oomycete pathogenicity is essential to gain the full context of how these pathogens cause disease on plants. An intense research focus on secreted *Phytophthora* effector proteins, especially those containing a conserved N‐terminal RXLR motif, has meant that most cell biological studies into *Phytophthora* diseases have focussed on the effectors and their host target proteins. While these effector studies have provided novel insights into effector secretion and host defence mechanisms, there remain many unanswered questions about fundamental processes involved in spore biology, host penetration and haustorium formation and function.

**Table:** 

**Contents**		
	Summary	445
I.	Introduction	445
II.	*Phytophthora* lifestyles; the knowns and the Introduction	446
III.	Pathogenicity factors enable infection	449
IV.	Pathogenicity factor delivery	451
V.	Host cell components reorganise to facilitate defence	452
VI.	Mysterious haustoria; the known unknowns	453
VII.	Conclusion and outlook	454
	Acknowledgements	454
	References	454

## I. Introduction

*Phytophthora* species are oomycetes, which are classed as stramenopiles (or heterokonts) and are thus related to brown algae, but have filamentous fungus‐like growth habits. All species are plant pathogens and some cause serious diseases in important crops, including some of the world’s favourite foodstuffs: chocolate, soy, tomato and potato. In addition, they can devastate forests, causing major losses to the forestry industry, damaging the natural environment and threatening to derail attempts to mitigate climate change.

Among the oomycetes, the *Phytophthora* species are some of the best studied due to the magnitude of their economic impact and because their hemibiotrophic, and in some cases also saprophytic (Hardham, 2005; Aram & Rizzo, 2018), lifestyles allow them to be cultured.

The pathogenic lifestyle is fundamentally about feeding from a host, and the success or failure of an infection is entirely dependent on the ability of the pathogen to overcome host defences. Most plants are resistant to most pathogens and, even on susceptible hosts, many infection attempts fail. The plant defence system is sophisticated and complex, involving many different signalling pathways and extensive cross‐talk between them. It has traditionally been described as a roughly two‐tiered system, with the initial responses, called pattern‐triggered immunity (PTI), being to conserved molecular patterns produced by microbes or generated through their interactions with the plant. The second layer, effector‐triggered immunity (ETI), involves recognition of, and responses to, pathogen effector molecules by receptors (Jones & Dangl, 2006). Y. Wang *et al*. (2019) propose an updated and more integrated model that describes plant–pathogen interactions as a three‐layered system, with a recognition layer, a signal‐integration layer and a defence‐action layer.

## II. *Phytophthora* lifestyles; the knowns and the unknowns

### 1. Getting there and getting in

**Fig. 1:**
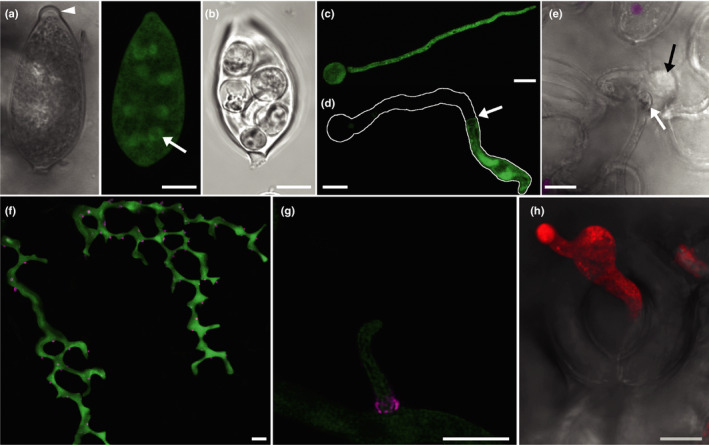
Confocal images of *Phytophthora infestans* growth stages. (a) A single optical section of the papillate sporangium of a GFP‐expressing *P. infestans* transformant is shown in false transmission and fluorescence modes. The papilla is indicated by the arrowhead. The nuclei are visible in the latter as circles of GFP fluorescence (green); one is arrowed. (b) A sporangium that has undergone zooporogenesis shown in false transmission. (c) A projection image of a germinated cyst of a GFP‐expressing transformant. (d) A germinated cyst in which the germ tube cytoplasm has concentrated towards the growing tip, presumably as a result of the formation of a plug at the point indicated with an arrow. The outline of the whole germinated cyst is traced in white. (e) A germ tube that has penetrated a cell adjacent to a stomate to form an infection vesicle (black arrow). The swollen end of the germ tube is indicated with the white arrow. Chloroplast autofluorescence is overlaid onto the false transmission image in magenta. (f) A projection image of infectious hyphae of a transformant expressing GFP in the cytoplasm and mRFP fused to the effector Avr3a (magenta), which is mainly secreted at haustoria. The image shows the stepwise pattern of growth that occurs between the upper leaf epidermis and the palisade mesophyll. (g) A magnified, deconvoluted image of a haustorium expressing Pi04314‐mRFP that has the most intense red fluorescence around the base, indicating potentially the highest level of secretion in this zone. (h) A projection image of a sporangiophore of a transformant expressing tdTomato fluorescent protein (red) emerging from an open stomate which is shown in the false transmission overlay. Bars, 10 µm.

*Phytophthora* dispersal forms may be multinucleate sporangia (Fig. 1a), zoospores or long‐lived oospores and chlamydospores, although these latter two do not so much disperse as lie in wait for favourable conditions. Caducous species release their sporangia for dispersal by wind and rain. Noncaducous species rely on zoospores for independent movement of inoculum. Surprisingly, given their lack of reinforcing cell walls, the zoospores of some species can apparently survive in moist environments for extended periods (e.g. Declercq *et al*., 2012). Physical transport of spores in soil and infected material by animals, farming and nursery practices, imports and exports, or extreme weather events will also distribute inoculum. Sporangia either germinate directly, which, for *P. infestans*, is favoured at higher temperatures (Judelson & Blanco, 2005), or differentiate (Fig. 1b) into swimming biflagellate zoospores, which are released through an apical pore. Depending on the species, sporangia may be papillate (Fig. 1a) or nonpapillate. Germination or zoospore release occurs at the opposite end to the point of attachment to the sporangiophore. Zoospores are attracted to both chemical and electrical signals from hosts (Judelson & Ah Fong, 2019). Once they have reached the host surface they encyst and adhere, then commence germination (Fig. 1c) in as little as 20 min (*P. cinnamomi*; Hardham, 2005). They are generally observed to germinate rapidly, although *P. infestans* cysts may take up to 2 h to germinate, and *P. ramorum* cysts may remain dormant for 2 d (Moralejo & Descals, 2010). As encystment and germination will occur in water, these processes do not absolutely require host components, although exposure to host compounds stimulates germination (Judelson & Ah Fong, 2019).

**Fig. 2:**
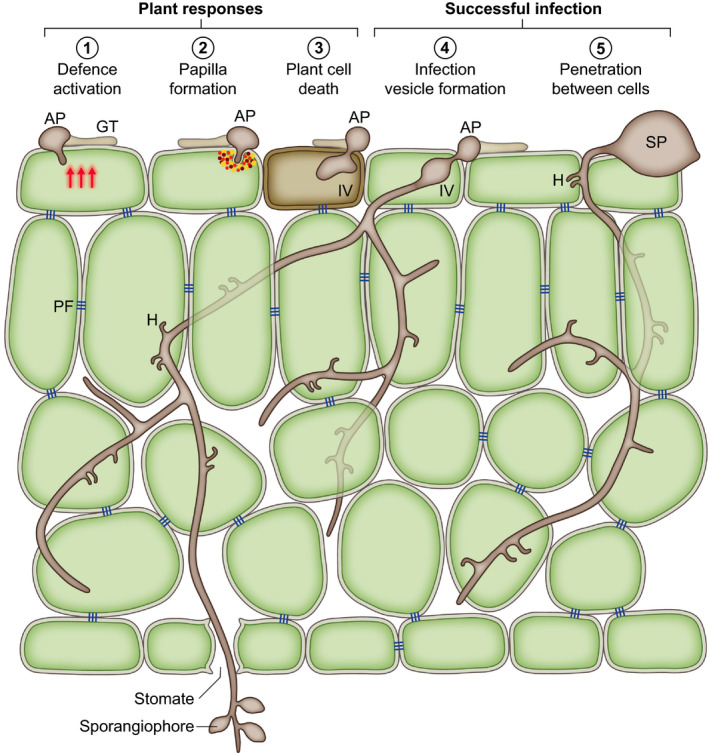
*Phytophthora* infection processes; schematic drawing of a section of a leaf invaded by *Phytophthora*. Germination of a sporangium (SP) or cyst on the leaf surface produces a germ tube (GT), which may form an appressorium (AP) (1–4) or may penetrate between anticlinal walls (5). Host penetration triggers plant defence responses (1). These may result in the formation of a papilla (2) or the death of the initially infected cell (3) which can prevent further infection. Host penetration may involve the formation of an infection vesicle (IV) (3, 4), an expanded intracellular structure, from which infectious intercellular hyphae extend to ramify through the plant tissue. Intercellular growth patterns suggest that *Phytophthora* avoids disrupting pit fields (PF); groups of plasmodesmata that connect plant cells. During biotrophic growth haustoria (H) are formed in cells contacted by the hyphae. Sporangiophores commonly emerge through stomata in leaf infections.

Germ tubes grow over the host surface and may form an appressorium‐like swelling when a suitable site for host entry is located (Fig. 2). Appressoria will form on inert surfaces (e.g. Kots *et al*., 2017) so there are no host factors that are essential for their development. However, there may be stimuli or conditions on host surfaces that encourage their formation. For example, Wang *et al*. (2012) demonstrated that cutin monomers increased *P. palmivora* appressorium formation by seven‐fold on a synthetic surface. Penetration of the host may also occur through natural openings such as stomata (e.g. *P. palmivora*; Sarria *et al*., 2016) or lenticels (e.g. *P. infestans* entry into potato tubers; Judelson & Ah Fong, 2019, *P. ramorum* entry into stems; Oßwald *et al*., 2014), directly between anticlinal walls of surface cells without appressorium formation (e.g. *P. cinnamomi*; Hardham, 2005, O’Gara *et al*., 2015; Fig. 2) or through wounds. Curiously *P. infestans* appears to avoid entering leaves through stomata; cyst germ tubes can be observed growing across stomata (e.g. Grenville‐Briggs *et al*., 2008), instead preferring trichomes and locations close to cell boundaries (Avrova *et al*., 2007). O’Gara *et al*. (2015) also noted that *P. cinnamomi* rarely entered through stomata.

*Phytophthora* appressoria are small compared with fungal appressoria, and we observed that they are not uniform in size or shape, and are unpigmented. They are assumed to develop turgor pressure, but the pressure may be much lower than the very high pressure generated by *Magnaporthe* species, as measured by Howard *et al*. (1991). *Phytophthora* species may therefore rely more heavily on enzymatic degradation of the plant cuticle and wall. In agreement with this, numerous copies of cutinase‐encoding genes are present in *Phytophthora* genomes, and their expression peaks at the early stages of infection (Muñoz & Bailey 1998; Ospina‐Giraldo *et al*., 2010). Kots *et al*. (2017) showed that an actin aster is formed in *P. infestans* appressoria at the host penetration point, but it does not resemble the actin ring observed in *Magnaporthe oryzae*. They propose that the actin aster enhances vesicle transport to the developing penetration point rather than the creation of a diffusion barrier, as in *M. oryzae* (Dagdas *et al*., 2012). Kots *et al*. (2017) also show that the septa‐like structures, known as plugs, which are composed of cell wall material and associated with cytoplasmic retraction towards the growing tip (Fig. 1d), form in germ tubes originating from cysts but not sporangia. These may thus be more for conservation of limited resources rather than to assist the generation of turgor.

Once the plant cell has been successfully penetrated an infection vesicle may form inside the first invaded cell (Fig. 1e) and, from this, infectious hyphae may ramify through the tissue in multiple directions (Avrova *et al*., 2007; Fig. 2). Infection via other routes such as stomata may not involve infection‐vesicle‐like structures, just hyphal growth (Dale & Irwin, 1991). Successful infection is not initiated by every zoospore or sporangium that encounters a host plant (Leesutthiponchai & Judelson, 2019) and many of the notorious *Phytophthora* species may be successful by virtue of the production of very large numbers of sporangia and zoospores. Attempted penetrations may be thwarted by the formation of a papilla by the host (Fig. 2), an accumulation of cell wall carbohydrates, such as callose and arabinogalactan, phenolic compounds and proteins (Collinge, 2009). Even if the infection has progressed to the point of penetration or the formation of an infection vesicle, the infected cell may undergo cell death before the infection can progress (Fig. 2). This cell suicide is a key strategy in plant defence against biotrophic and hemi‐biotrophic pathogens (Dickman & Fluhr, 2013). The initiation of infection is a race between pathogen and host, with the pathogen trying to tip the balance towards suppression of defence and get beyond the initially infected cell, while the host tries to launch and complete defence responses before it does so.

### 2. The progression of infection

If the infection is successfully established, hyphae subsequently grow intercellularly (Fig. 2). From our observations of *Phytophthora* species in leaves, it appears that they avoid disrupting pit fields, leading to a stepped pattern of hyphal growth at the top of the palisade mesophyll (Fig. 1f and Whisson *et al*., 2007). As hyphae grow through the plant tissue, they extend haustoria into host cells, locally digesting the cell walls to allow the haustoria through (e.g. Shimony & Friend, 1975) (Figs 1f,g, 2). Haustoria are thus brought into close contact with the host membrane to facilitate efficient delivery of defence‐controlling pathogenicity factors and perhaps nutrient uptake. The *P. infestans* haustorium is a major site of protein secretion during infection, including cytoplasmic and apoplastic effectors and cell wall degrading enzymes (Wang *et al*., 2018). Judelson & Ah Fong (2019) suggest, however, that haustoria are not the major sites of nutrient acquisition for the hemibiotrophic *Phytophthora* species due to a lack of nutrient transporters specific to haustorium‐forming species, the lack of haustorial neckbands and that haustoria only represent about 2% of the pathogen surface area. Thus, their primary role may be defence suppression.

When the infection has progressed sufficiently, sporangiophores are extruded. In leaf infections this occurs predominantly through stomata, although also simply between epidermal cells (Figs 1h, 2). Sporangiogenesis in root‐infections occurs either at surface roots, to enable spore dispersal by rainwater, or simply out of roots into the surrounding soil/rhizosphere (Judelson & Ah Fong, 2019). In stem infections sporangiophores may emerge through lenticels on stems (Oßwald *et al*., 2014). Sporangiophore emergence on leaves generally occurs at night, this strategy has the advantage that the sporangia then have reduced exposure to UV radiation. Interestingly this must involve manipulation of stomatal regulation (Judelson & Ah Fong, 2019), presumably by pathogenicity factors produced later in the infection time course. *Pseudomonas syringae* is known to produce the toxin coronatine, which mimics conjugated jasmonic acid and induces stomatal opening (Melotto *et al*., 2006) but there are other pathways a pathogen could manipulate to achieve this end (reviewed by McLachlan *et al*., 2014).

*Phytophthora* species are typically considered to be hemibiotrophic in that the initial phase of infection involves both the production of haustoria and keeping the host cells alive. Maintenance of this phase requires continual suppression of defences in each newly penetrated cell. After this, the infection switches to a necrotrophic mode in which host cells die and are digested. This has been suggested to be a deliberate process of cell killing by upregulated expression of, for example, cytotoxic necrosis and ethylene‐inducing peptide 1 (Nep1)‐like proteins (NLPs) at the onset of the necrotrophic phase (e.g. Dong *et al*., 2012). Deliberate necrotrophy is also supported by the finding that the expression of metabolic enzymes by *P. infestans* in the necrotrophic stage became more similar to that of *Pythium ultimum*, a necrotroph (Ah‐Fong *et al*., 2017). The length of the biotrophic phase depends on the pathogen species and the host. For *P. infestans* on potato gene expression, analyses indicate that the biotrophic phase is the first 3‐ to 4‐d postinfection (Whisson *et al*., 2007; Haas *et al*., 2009; Cooke *et al*., 2012) and that the necrotrophic phase starts after that. For *P. capsici* on tomato, the switch to the necrotrophic phase is as early as 1 d (Jupe *et al*., 2013). Timelines of the infection stages based on macroscopic observations or gene expression analyses of homogenised samples, however, do not always correlate well with the behaviours observed at the microscopic scale. We observe with *P. infestans* that the germination of inoculum is not synchronous and, while zoospores and cysts generally have a limited lifespan in contact with the host, it can be days before an individual sporangium germinates on a leaf surface. Thus the timeline for that individual does not correlate well with the ‘hours post inoculation’ of the inoculum as a whole. The germ tubes from sporangia can grow quite extensively before they form appressoria/penetrate (or die). Germ tubes from cysts conversely have a more limited source of cytoplasm and energy as cysts are smaller than sporangia and thus may not grow as extensively. The time from germination to appressorium formation/penetration can therefore be quite variable. In an infection growing radially, the leading edge may continue to display biotrophic behaviour for many days beyond the point at which the central zone has become necrotic.

Descriptions of infection processes and phases are largely based on studies of infections in accessible and readily manipulated rapidly growing plants. Less has been described about the cell biology of the development and spread of infections in tree species, especially in the woody tissue. Although most tree pathogens are described as root pathogens, O'Gara *et al*. (2015) showed that *P. cinnamomi* entry into stems occurred at sites where the periderm was thin, and wounding was shown to be a key factor for *P. ramorum* infection of stems (Tooley *et al*., 2014). Infections initiated in the roots are also known to spread upwards into the trunks of trees and there is some evidence that this occurs via the xylem (Brown & Brasier, 2007). In what form and manner *Phytophthora* infection moves through the xylem and details of its entry into and emergence from there in woody hosts have not been resolved.

## III. Pathogenicity factors enable infection

The best studied *Phytophthora* species are known to secrete hundreds of pathogenicity factors to enable the establishment and development of infection. This seems reasonable considering the complexity of the plant immune system. However, other pathogenic organisms, such as bacteria, successfully establish infections with far fewer. Thordal‐Christensen *et al*. (2018) tackled this conundrum and suggest that the developmental complexity of filamentous pathogen infections may explain it. In plant pathogens the best studied pathogenicity factors are proteins and these can be divided roughly into those that act in the apoplast and those that are translocated into host cells, which are referred to as cytoplasmic effectors. Much less information has been published about nonprotein effectors as Collemare *et al*. (2019) commented with their review title ‘Nonproteinaceous effectors: the terra incognita of plant–fungal interactions’. They point out that the activities of many small molecules derived from plant pathogenic fungi have provided cell biologists with inhibitors that are very useful for studying cellular processes. Thus these molecules are likely to enable the pathogens to interfere with defence responses. Moreover, research into RNA‐based pathogenicity factors has seen a recent upsurge of activity. Cross‐kingdom RNAi has been clearly shown with recent discoveries in a variety of plant–microbe interactions (e.g. review by Cai *et al*., 2019). It is early days for the characterisation of small RNAs produced by, or targeted to, *Phytophthora* species but it is evidently important, as a *P. capsici* effector has been found to specifically suppress the production of small RNAs in Arabidopsis that were detected in extracellular vesicles (EVs) and appear to target pathogen genes (Hou *et al*., 2019). It has also been suggested that mRNAs might be translocated to cause the production of pathogen‐encoded proteins in host cells, although this has yet to be shown. It would be interesting to determine whether there is trafficking of plant‐targeted RNA species to haustoria.

### 1. Apoplastic effector proteins

Apoplastically active proteins produced by *Phytophthora* species were the first pathogenicity factors to be studied. One of the first to be cloned was the CRY‐B elicitin (Panabières *et al*., 1995) (an elicitin is a protein that elicits a defence response from plants). Elicitins are a conserved class of apoplastic proteins thought to be involved in sterol scavenging (although some bind to other lipids) and may be recognised as microbe‐associated molecular patterns (MAMPs) by plant defence receptors (reviewed by Derevnina *et al*., 2016). The *P. infestans* elicitin INF4, however, does not trigger cell death *in planta*. Moreover, its transient expression in *Nicotiana benthamiana* led to enhanced pathogen colonisation, suggesting that this elicitin was potentially a virulence factor. Transgenic *P. infestans* that expressed INF4‐mRFP revealed that INF4 is secreted from haustoria during infection (Wang *et al*., 2018).

Small cysteine‐rich (SCR) secreted proteins, such as SCR74 (Liu *et al*., 2005) and the related PcF (Orsomando *et al*., 2001), form another class of apoplastic factors and have been described from several *Phytophthora* species. Some have been shown to cause cell death when applied to plants. However, as with the elicitins, the cell death responses may be due to their recognition as MAMPs (Nie *et al*., 2019) and by triggering of a hypersensitive response rather than any specific necrotising activities of the proteins themselves. Their actual functions in the infection process have not been resolved, although X. R. Chen *et al*. (2016) showed that *P. cactorum* lines silenced for SCR96 were more susceptible to oxidative stress, thus suggesting a role in protecting *Phytophthora* species from plant defensive reactive oxygen species.

The NLP family of apoplastic effectors is expanded in *Phytophthora* species (e.g. Dong *et al*., 2012). They can be separated into two functional classes: cytolytic (cNLPs) and noncytolytic (ncNLPs; e.g. Cabral *et al*., 2012; Oome & Van den Ackerveken, 2014; Lenarčič *et al*., 2017, 2019). Thus, at least for the cNLPs, the specific activity of these apoplastic factors causes cell death, although NLPs can also be recognised as MAMPs (Oome *et al*., 2014). Interestingly the cytolytic members are generally only cytolytic on eudicots, due to the specificity of their binding to series A glycosylinositol phosphorylceramide (GIPC) sugars, which decorate sphingolipids in the plasma membranes of eudicots but not generally monocots (Lenarčič *et al*., 2017). The cNLPs are not produced by the obligately biotrophic oomycete *Hyaloperonospora arabidopsidis* (Cabral *et al*., 2012). They tend to be expressed later in infection by the hemibiotrophic *Phytophthora* species, and thus are associated with the switch to the necrotropic phase (e.g. Qutob *et al*., 2002; Kanneganti *et al*., 2006). The function of the ncNLPs produced by *Phytophthora* species early in infection has not been resolved.

Proteases are key elements of plant defence (e.g. Thomas & van der Hoorn, 2018) and thus it is no surprise that *Phytophthora* species have evolved an arsenal of protease inhibitors. The first characterised were Kazal‐like serine protease inhibitors from *P. infestans* (Tian *et al*., 2004). This was followed by the identification of cystatin‐like EPICs (Tian *et al*., 2007). The substrate specificity of the EPIC1 variants of the sister species *P. infestans* and *P. mirabilis* was suggested to be important for their host restriction (Dong *et al*., 2014). A degree of host specificity in the protease inhibitor suite may also occur in *P. palmivora*. This brutal pathogen is not deterred by the high levels of papain in papaya due to the efficiency of one of its cysteine protease inhibitors PpaEPIC8 (Gumtow *et al*., 2018) whereas, for its infection of rubber trees, the Kazal‐like serine protease inhibitor PpEPI10 may be more important (Ekchaweng *et al*., 2017).

Small phospholipase D (PLD)‐like proteins have been shown to be virulence factors for *P. infestans* (Meijer *et al*., 2018). Some possess signal peptides while others do not. Meijer *et al*. (2018) found that transient *in planta* expression of three PLD‐like proteins enhanced *P. infestans* infection. For the two signal‐peptide‐containing PLD‐like proteins tested this was dependent on the signal peptides being present. Whether the PLD‐like proteins without signal peptides are also secreted and whether the PLD‐like proteins function in the apoplast or are translocated into the host is unresolved but, given that PLD activity was recovered from growth medium of cultured *P. infestans* (Meijer *et al*., 2014), at least some of them probably function in the apoplast. PLD activity generates phosphatidic acid (PA), which has been found to promote membrane curvature and is possibly involved in promoting vesicle fusion (Zhukovsky *et al*., 2019). Potential roles for PLDs in the *Phytophthora*–host interface suggested by this property of PA may be for generation of membrane curvature during host cell penetration and folding of membranes that have expanded to increase host–pathogen exchange, for example.

Genes encoding cyclophilins, peptidyl‐prolyl isomerases involved in protein folding, posttranslational modification and protein–protein interactions, are well represented in *Phytophthora* genomes, although only a small number appear to be upregulated during infection (Gan *et al*., 2009). One of these, PnCyPA, lacks a signal peptide but was shown by immunofluorescence to be secreted to the surface of *P. nicotianae* germinated cysts (Gan *et al*., 2009). The roles of cylophilins as pathogenicity factors have been demonstrated in a few phytopathogenic fungi through gene knockouts (reviewed by Singh *et al*., 2018). Their mechanisms of action in pathogenicity, however, have not been resolved. Gan *et al*. (2009) suggested that the secreted cyclophilins in *Phytophthora* may play a role in the folding and activation of apoplastic effectors, implying that they are not themselves effectors. Similarly, a secreted protein disulphide isomerase of *P. parasitica* (PpPDI1) tagged with GFP was found to localise to haustoria (Meng *et al*., 2015). PDIs are involved in disulphide bond formation, breakage and rearrangements and are thus key protein folding enzymes. PpPDI1 was difficult to silence, which suggests that it is essential to *P. parasitica* (Meng *et al*., 2015). Its overexpression increased pathogenicity and the number of haustoria formed.

Cell wall degrading and modifying enzymes (CWDEs) are key elements of the *Phytophthora* infection process as they are involved in entry into the host, hyphal ramification through the apoplast, and the formation of haustoria. Micrographs of appressoria and haustoria show that the plant cell wall is degraded around the plant cell penetration points and between the haustoria and plant plasma membrane (e.g. Shimony & Friend, 1975). Silencing of CWDEs has been shown to reduce pathogenicity (e.g. Ma *et al*., 2015; Lai & Liou, 2018). CWDE families are expanded in *Phytophthora* species compared with *Pythium ultimum* (Brouwer *et al*., 2014; Yang *et al*., 2018). A broad range is expressed during infection but different family members may be expressed in any given lifecycle stage, host or tissue‐type (Attard *et al*., 2014; Blackman *et al*., 2015). In addition to removing the plant cell walls at these penetration points, it is possible they may also modify the structure of the *Phytophthora* cell walls at these intimate contact points to allow more efficient exchange with host cells.

### 2. Translocated effector proteins

*Phytophthora* researchers got a lucky break when the first avirulence proteins from oomycetes were compared, revealing a conserved Arg–any residue–Leu–Arg motif (RXLR), often accompanied by a Glu–Glu–Arg motif (EER) shortly downstream (Rehmany *et al*., 2005). Analyses of sequenced *Phytophthora* genomes for putative secreted proteins has revealed the RXLR–EER motif in hundreds of sequence‐diverse small secreted proteins (McGowan & Fitzpatrick, 2017). The RXLR family of effectors contains all the known cytoplasmic avirulence (Avr) proteins for *Phytophthora* species and downy mildews (Y. Wang *et al*., 2019). ATR5 from *H. arabidopsidis* does not have a typical RXLR motif but does contain an appropriately located EER, and thus may be in the same family (Bailey *et al*., 2011). A common but not ubiquitous structural motif in RXLR effector domains is the WY motif and some RXLRs have a degree of modularity to their effector domains with repeats of WY or LWY structural motifs (Jiang *et al*., 2008; Win *et al*., 2012; He *et al*., 2019).

Another group of putative effectors with a clear motif in the amino‐terminal region (LFLAK) was named the crinkler and necrosis family (CRN) after the crinkling and cell death phenotypes observed when some of the initially discovered genes were expressed *in planta* (Torto *et al*., 2003). The cell death phenotype has turned out not to be a particularly diagnostic feature of the family (Stam *et al*., 2013). While RXLR effectors generally have a roughly bipartite structure of RXLR(–EER) domain plus a C‐terminal effector domain, CRNs appear to be modular and composed of a variety of domains in different orders (Haas *et al*., 2009; Stam *et al*., 2013).

Schornack *et al*. (2010) showed that the putative CRN translocation motif fused to the *P. infestans* Avr3a C‐terminal domain resulted in a loss of colonisation on plants expressing the R3a receptor, but no direct observation of the translocation has been published. CRNs have been shown to mostly localise in the nucleus when they are expressed *in planta* (Schornack *et al*., 2010; Stam *et al*., 2013) but little information has been published to resolve their functions. Song *et al*. (2015) showed binding of a *P. sojae* CRN to plant heat shock protein (HSP) promoters and Zhang *et al*. (2015) showed that two *P. sojae* CRNs interacted with catalases and, curiously, had opposing effects on hydrogen peroxide accumulation.

**Fig. 3:**
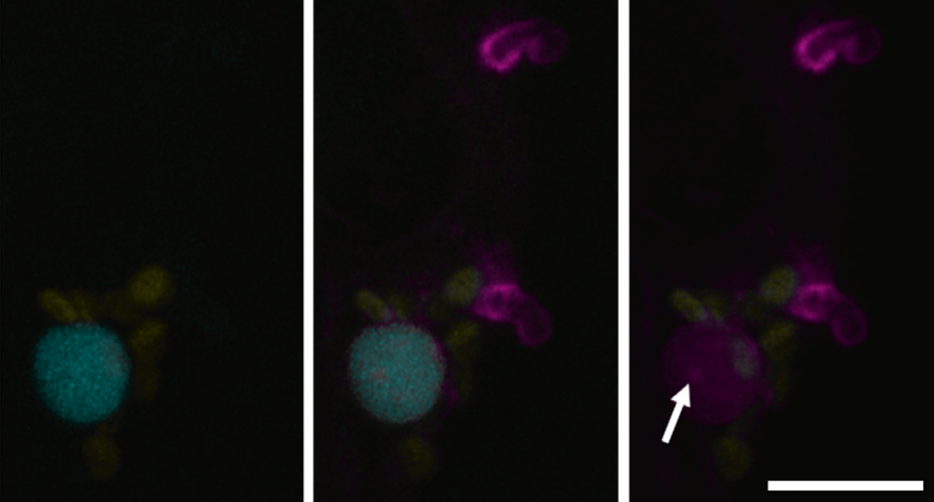
Confocal image showing the translocation of a nuclear‐targeted RXLR effector. Infection of a transgenic plant expressing a CFP‐histone 2B fusion (cyan) by a *Phytophthora infestans* transformant secreting RXLR effector Pi22926‐mRFP fusion protein from haustoria. mRFP fluorescence (magenta) is detectable in the nucleus and nucleolus (arrow) of haustoriated cells indicating translocation has occurred. Chloroplast autofluorescence is shown in yellow. Bar, 10 µm.

The initial evidence for translocation of RXLR and CRN effectors inside plant cells was based on outputs from indirect assays such as plant resistance initiated by cytoplasmic resistance proteins (Armstrong *et al*., 2005; Whisson *et al*., 2007; Dou *et al*., 2008; Schornack *et al*., 2010). However, we now have definitive evidence that the RXLR effectors are indeed translocated from haustoria into infected plant cells (Fig. 3 and S. Wang *et al*., 2017). This is something of a relief as many researchers have spent a decade characterising RXLR effector functions *in planta*. There have been several recent reviews about the functional characterisation of RXLR effectors (e.g. Whisson *et al*., 2016; Y. Wang *et al*., 2019; Wang & Fangchan 2019). *In planta* expression of fluorescent protein tagged *P. infestans* RXLR effectors reveals that they locate to many subcellular organelles and structures (S. Wang *et al*., 2019). What is clear from the functional studies performed is that RXLR effectors target a wide range of pathways throughout the plant cell, often in multiple redundant ways. *P. infestans* RXLR effector Pi22926 and PexRD2, for example, both suppress the Cf4/Avr4 cell death response but by targeting different MAP3Ks in the signalling cascade (King *et al*., 2014; Ren *et al*., 2019). PiAvr3a also supresses the Cf4/Avr4 cell death response through its action on CMPG1 (Gilroy *et al*., 2011). A key role for the effector suite is the suppression of pathogen‐associated molecular pattern (PAMP) response pathways (i.e. PTI). For example, several were found to suppress the responses induced by the essential CWDE XEG1 (Ma *et al*., 2015), but some have also been shown to inhibit recognition responses to other RXLR effectors and are thus acting on the ETI responses (Wang *et al*., 2011; H. Wang *et al*., 2017).

RXLR effectors have been shown to have a variety of effects on their host target proteins, one of which is altering their localisation. *P. sojae* effector PsAvh52 relocates an acetyltransferase GmTAP into the nucleus where the latter acetylated histones (Li *et al*., 2018). This resulted in upregulation of the expression of genes that increased susceptibility (Li *et al*., 2018). The *P. infestans* effector Pi04314 relocalised the protein phosphatase 1 catalytic subunit from the nucleolus to the nucleoplasm, where it presumably dephosphorylates proteins that play a role in host defence (Boevink *et al*., 2016). By contrast Pi03192 prevented relocalisation of membrane‐localised NAC transcription factors to the nucleus in response to defence stimulation (McLellan *et al*., 2013) and PiAvrblb2 prevented secretion of the C14 protease (Bozkurt *et al*., 2011). It will be interesting to learn how Avrblb2 achieves this, perhaps it involves stimulating the release of the protease into the cytoplasm from the endoplasmic reticulum (ER), which Bozkurt *et al*. (2011) noted was reported to happen during drought stress (Harrak *et al*., 2001). Another activity shown by several effectors is modulation of target protein stability. Yang *et al*. (2019) demonstrate that *P. sojae* effector PsAvh238 interacts with and destabilises GmACS1, an enzyme involved in ethylene biosynthesis. Pi02860 uses NRL1 to destabilise the guanine nucleotide exchange factor SWAP70 (He *et al*., 2018). Conversely Li *et al*. (2019) show that *Phytophthora* effectors related to PiAvr3a stabilise cinnamyl alcohol dehydrogenase (CAD)‐subfamily 7 proteins. CAD family proteins are involved in lignification, which is part of plant structural defences (e.g. Tronchet *et al*., 2010 *)*, and it would thus seem counterintuitive that effectors might enhance their activity. Li *et al*. (2019) found, however, that the CAD7 family had little impact on the lignin content and their enzymatic activity was not involved in their ability to enhance susceptibility of Arabidopsis to *P. capsici* infection. Given that PiAvr3a stabilises the E3 ligase, CMPG1 (Bos *et al*., 2010), Li *et al*. (2019) tested whether it interacted with the *N. benthamiana* homologue of CAD7. No direct interaction was detected with the methods used, so CAD7 may not be a target of CMPG1.

While there are still very many effectors to be studied, some groups are now looking further into the pathways targeted by effectors, such as autophagy (Zess *et al*., 2019), illustrating that effectors are indeed useful tools to probe plant defence responses.

## IV. Pathogenicity factor delivery

**Fig. 4:**
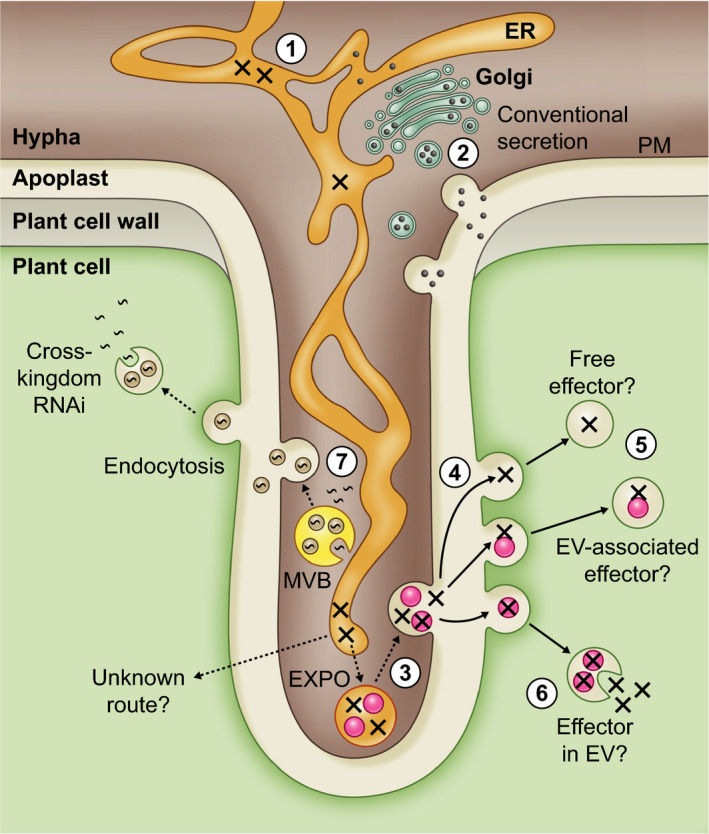
Schematic drawing of a plant cell invaded by a haustorium illustrating routes for pathogenicity factor delivery. Apoplastic proteins (black circles) are secreted via the conventional secretory pathway (1). This involves entry into the endoplasmic reticulum (ER) followed by passage through the Golgi apparatus and sorting into secretory vesicles which fuse to the *Phytophthora* plasma membrane (PM) (2). The sequences of RXLR effectors (crosses) include signal peptides and thus they also enter the ER. Evidence from two RXLR effectors indicates that they are then secreted by a different, unknown route and are translocated into host cells. One possibility might be that they are sorted into a distinct domain of the ER (3) which may then cleave off from the rest of the ER, perhaps as an exosome positive organelle (EXPO). This is an autophagosome‐like body that is thought to form by the ER extending, curling back on itself, and engulfing cytoplasm (Wang *et al*., 2010). The EXPO may then fuse with the PM. In what form the RXLRs exist in the apoplast is unknown. They may be free proteins, in protein complexes, or associated with extracellular vesicles (EVs). They may be associated with the exterior, presumably proteinaceous coat, of the EVs or inside them. How the latter might occur is unknown. Once released into the apoplast the RXLR effectors could be taken into host cells by endocytosis (4). If the RXLR effectors are taken up as proteins or associated with the exterior of EVs, how they then exit the endosomes to access the plant cell interior is unknown (5). If they were inside EVs then the EVs could potentially fuse with the endosome membranes and thereby release their contents (6). Cross‐kingdom delivery of RNA molecules has been shown to involve EVs (7) (Buck *et al*., 2014; Cai *et al*., 2018). The RNAs could be taken up from the cytoplasm of the pathogen by invagination of the outer membranes of multivesicular bodies (MVBs). The MVBs then fuse with the PM to release RNA‐carrying EVs which can be endocytosed by the host cells. As suggested for RXLR effectors the RNA could be released into the host cell by fusion of the endocytosed EVs with the endosome membrane.

While apoplastic effector proteins simply require secretion into the apoplastic space through conventional secretion (Fig. 4), factors that function within host cells must have some means of crossing the pathogen–host divide unmolested and then entering the host cell. Our work on two RXLR effectors indicated that they are secreted from *Phytophthora* by a different pathway than apoplastic effectors (S. Wang *et al*., 2017; Wang *et al*., 2018). It is not known whether this is the case for all cytoplasmic effectors. How they exist in the extracellular environment and how they enter host cells to reach their sites of activity has also not been resolved. The work described by Kale *et al*. (2010) implicated phosphatidylinositol‐3‐phosphate (PI3P) in RXLR effector uptake by plant cells. However, this has been challenged (e.g. Wawra *et al*., 2012; Boddey *et al*., 2016). There is evidence from work on a few different RXLR effectors that the RXLR motif is cleaved within the pathogen before secretion, which is incompatible with it playing a role in cell entry (Wawra *et al*., 2017; S. Wang *et al*., 2017; Wang *et al*., 2018; Schoina *et al*., 2019). It has been suggested that PI3P may play a role in a sorting mechanism within the pathogen secretory system and expression of a PI3P sensor in *P. sojae* showed that it appeared to be enriched at haustoria (L. Chen *et al*., 2016). While secretion mechanisms such as those involving EVs have yet to be characterised in *Phytophthora*, it is tempting to speculate that the effectors may end up in, or be associated with, EVs (Fig. 4). How that might happen and what class of EVs might be involved can only be guessed at, as most classes of EVs described are associated with secretion of proteins that do not contain signal peptides (discussed in Boevink, 2017). For host cell entry the most straightforward route would involve endocytosis. However, how effectors would then exit from endocytic compartments, especially if they are not in EVs, is more difficult to envisage. Endocytosed EVs could fuse to endocytic compartment membranes to release their contents into the cytoplasm.

RNAs are notoriously labile and must be protected from attack. It has been shown that small RNAs targeted to pathogens are present in EVs (Cai *et al*., 2018; Hou *et al*., 2019). We assume that *Phytophthora* species also produce small RNAs to target host genes and these would also be packaged into EVs (Fig. 4). EVs would be relatively straightforward to isolate from *Phytophthora* species grown in culture. However, if the RNAs are only expressed during infection then they will be missed. Any *Phytophthora* EVs produced in the early biotrophic stages of plant infection are likely to be a very small fraction of the total EVs present which will make their characterisation challenging.

## V. Host cell components reorganise to facilitate defence

**Fig. 5:**
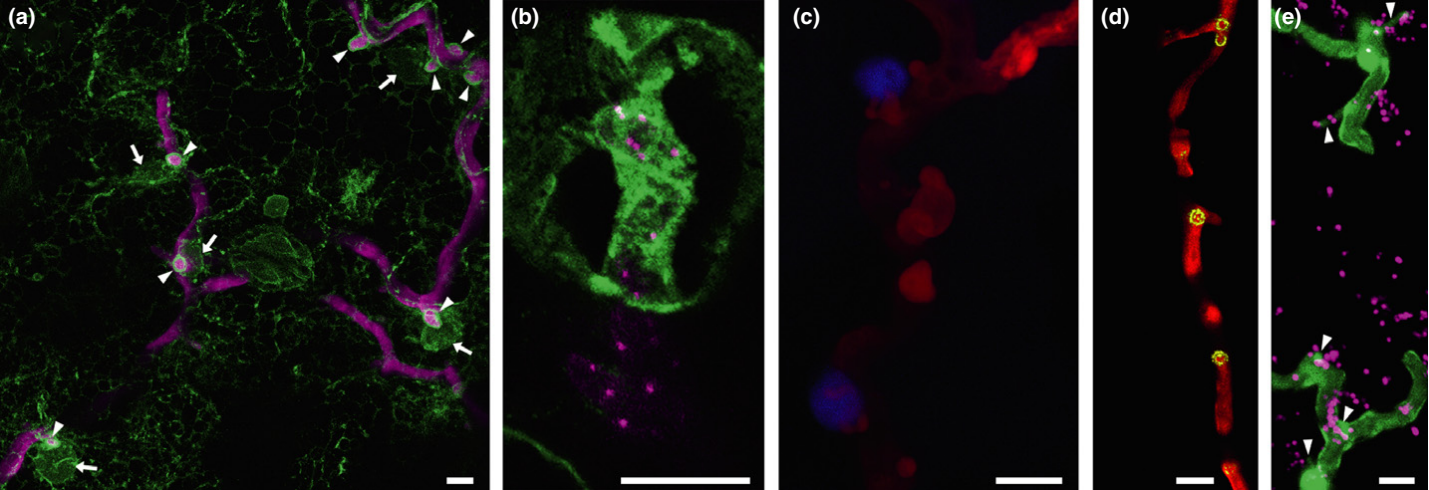
Confocal projection images showing the accumulation of cell components around *P. infestans* haustoria. (a) An image of the infection of a transgenic plant expressing GFP in the endoplasmic reticulum (ER; green) by a *P. infestans* transformant expressing the tdTomato fluorescent protein (magenta). Haustoria can be seen outlined in GFP‐labelled ER (arrowheads). Several of the haustoria have nuclei adjacent to them (arrows). (b) A higher magnification image of a haustorium from a tagRFP‐Lifeact transformant (in which actin plaques are evident as bright magenta spots) surrounded by GFP‐labelled host ER. (c) The association of nuclei with haustoria is more clearly visible in this image of a transgenic plant expressing a CFP‐histone 2B fusion (blue) infected with the same *P. infestans* transformant (red). Endosomes, such as those labelled by a YFP‐tagged exocyst subunit, Sec5 (d), and peroxisomes, labelled by mRFP‐SRL (in magenta in (e)) are also observed to cluster around haustoria. Haustoria are indicated by arrowheads in (e). Bars, 10 µm.

In addition to the many signalling pathways and defence reactions that occur at the molecular level (reviewed by Y. Wang *et al*., 2019), host cell components have been shown to relocate and reorganise in response to microbial attack. It is likely this reorganisation assists the speed and focus of the defence responses. The plant defence papilla, for example, is of no use if it is not constructed directly under the attempted penetration site. Takemoto *et al.,* (2003) showed actin reorganisation, microtubule (MT) depolymerisation, and ER (Fig. 5a) and Golgi body accumulation in Arabidopsis cells at oomycete penetration sites. Nuclei, peroxisomes, endomembrane compartments of the secretory pathway and exocyst subunits have also been observed clustering around haustoria (Fig. 5; Lu *et al*., 2012; S. Wang *et al*., 2019; Overdijk *et al*., 2020). Autophagosomes carrying Joka2 appear to be directed to the host–microbe interaction interface to play a role in defence, a role that is targeted by the RXLR effector RD54 which displaces Joka2 and thereby may change the cargo composition (Dagdas *et al*., 2016, 2018). A consequence of the heightened secretory activity around haustoria, and a degree of selectivity of the vesicle populations found there, is that the extrahaustorial membrane is distinct from the plasma membrane (Lu *et al*., 2012).

Nuclei have been observed to locate adjacent to haustoria in the early stages of cell infection (e.g. Schoina *et al*., 2017; Fig. 5a,b). This would presumably help to ensure a rapid deployment of responses derived from defence‐induced gene expression. There is increasing interest in the behaviour of chloroplasts during plant–microbe interactions. Chloroplasts play a central role in plant–microbe interactions and defence. They are involved in the synthesis of many defence molecules including reactive oxygen species and the key hormones salicylic and jasmonic acids (reviewed by Fernandez & Burch‐Smith, 2019). Toufexi *et al*. (2019) describe clustering of chloroplasts around haustoria. The chloroplasts were observed to extend stromules and wrap haustoria in a chloroplast–stromule network. There also appeared to be stromule links between multiple chloroplast clusters at different haustoria. Chloroplasts have also been shown to move to the nucleus after immune activation triggered by application of a viral protein; they moved along microtubules (MTs), led by stromule extensions, and appeared to be anchored at the nucleus by actin (Kumar *et al*., 2018). Combining these observations suggests the potential for a sequence of events in cells with a focal immune response to haustorial penetration; where chloroplasts might initially move to the nucleus and thus move to haustoria in association with the nucleus. Alternatively, the chloroplasts could locate to haustoria and assist the nucleus to move there through the development of stromule connections. However, in cells penetrated by multiple haustoria Toufexi *et al*. (2019) observed chloroplasts clustered around all of them. Given that there is only one nucleus per cell, it is more likely that chloroplasts move to haustoria independently of the nucleus. Takemoto *et al*. (2003) observed that there was an accumulation of diffuse fluorescence from GFP‐tagged tubulin around the haustorial penetration site, indicating a region of depolymerised MTs. This might suggest a restriction to chloroplast movement or that there might be an alternative movement mechanism to cross this zone. However, Takemoto *et al*. (2003) also noted that tubulin bundles could be seen running through the region of diffuse fluorescence.

Plant cells are well known to form papillae in response to microbial attack (Collinge, 2009) and, using similar expansions of cell wall materials, may encase haustoria. This is routinely observed with the biotrophic *H. arabidopsidis* (e.g. Lu *et al*., 2012; Caillaud *et al*., 2014) but rarely occurs with the aggressive hemibiotrophic *Phytophthora* species on susceptible hosts (e.g. Lu *et al*., 2012). Haustorial encasements were observed during *Phytophthora* infection of incompatible hosts (Enkeli *et al*.,^1997^; Lipka *et al*., 2005).

## VI. Mysterious haustoria; the known unknowns

Haustoria are involved in secretion of a range of pathogenicity factors (Wang *et al*., 2018). Although these pathogen projections into host cells are potentially vital for infection (Avrova *et al*., 2008), there are some crucial and rather gaping holes in our knowledge of basic haustorial biology. We outline some of them below. These holes exist in part due to the technical challenges in genetically manipulating *Phytophthora* species and also in part due to the limitations of live‐cell, high resolution imaging during infections.

Haustoria are not noted to form on hyphae grown in rich or defined growth media, but they are formed readily from intercellular hyphae during infection, and notably only where the hyphae contact plant cells. This raises the question of what are the defining signals and key factors that determine where a haustorium forms and whether it is successful or not. Cells can be penetrated by multiple haustoria, but does this only happen if they all form and penetrate simultaneously, or can they penetrate sequentially?

Entry of *Phytophthora* into plant cells occurs at the earliest stage of infection, by the penetration peg from appressoria, where they are formed, and the infection vesicle, and continues as the intercellular hyphae spread and come into contact with new host cells forming haustoria. Are there pathways and mechanisms involved in host penetration from an appressorium and formation of an infection vesicle that are also involved in haustorium formation?

*Phytophthora* species are hyphal organisms and, during infection, a disease lesion has an actively expanding front and behind this the host cells remain alive for a period before becoming necrotic. From the initial formation of a haustorium in a newly contacted host cell, how long does a haustorium remain functional in terms of delivery of the effectors that suppress defence responses? Is the longevity of haustorium function determined by the longevity of the cell after penetration and is this affected by: the type of cell (leaf, stem or tuber cell, cell type, size, activity, nutritional status, age), the number of haustoria that form in that cell, and the stage of the overall infection?

Studies using electron microscopy to describe the formation of haustoria have been invaluable in providing a static view into *Phytophthora* cellular organisation at these structures. However, infection and haustorium function suggest a highly dynamic cellular environment, and raises the question of whether cellular organelles are specifically trafficked to sites of haustorium formation and the speed at which this may occur. Furthermore, are the mRNAs that encode effectors specifically transcribed from the nuclei that are located closest to sites of haustorium formation?

When the infection front has moved on and infected cells begin to die, what happens to the haustoria that have finished their useful life? Is the whole structure dismantled and degraded or just left in place as effectively a small hyphal branch? If it is abandoned, is that by vacuolation or protoplast retraction? In what state is the plant cell when the haustorium is ‘finished’: already dead and degraded or just committed to death? Does the haustorium in fact play a key role in killing the cell by secretion of toxins or cytolytic proteins where the plant membrane is more accessible?

The types of experiments we can envisage to try to answer some of these questions require extensive amounts of time on microscopes, and high levels of skill, patience and persistence on the part of the experimenter. The limitations for live‐cell imaging of *Phytophthora* infections include plant tissue autofluorescence, especially from tissue that is damaged and dying due to the infection; the depth of the infection in the tissue, and the refractive properties of plant cell walls. These limit the ability to collect confocal images and particularly restrict the application of super‐resolution techniques. Time‐gated confocal microscopy was used by Overdijk *et al*. (2020) to separate GFP fluorescence and autofluorescence from plant defence responses by differences in their fluorescence lifetimes. Fluorescent protein tagging allows observations to be made over time in living tissue. However, illumination with UV or laser light could disrupt growth. We successfully monitored the emergence of a haustorium using a series of images collected *c*. every 3 h over several sites across a leaf, revealing that the initial entry of the haustorium into the plant cell occurred rapidly within 3 h (Avrova *et al*., 2008). Shorter time intervals between images would reveal haustorial growth in more detail but may be disruptive. If one subjects a haustorium to a fluorescent protein bleaching protocol it could prevent it from forming/growing/functioning any further or cause it to be abandoned. We did see recovery of tagged effector secretion within 4 h of bleaching (S. Wang *et al*., 2017). However, the conditions used to ensure sufficient bleaching, while allowing recovery, would need to be empirically determined for each experimental system.

Attempts have been made to develop alternative pathosystems for *Phytophthora* species, in part with a view to improving the accessibility of the infection structures to live‐cell microscopy. Gross *et al*. (1993) described the infection of parsley culture cells but this system did not gain any popularity in the field. More recently, tomato culture cells (Schoina *et al*., 2017) and moss (Overdijk *et al*., 2016) have been used. The moss system was only suitable for studying the very early general responses to penetration attempts, whereas the infections of the tomato cultures involved growth beyond the initially infected cell in clumps of cells and the formation of haustoria and is thus a superior system. Tomato cells still have walls, and thus their refractive properties will still confound microscopists, and the infections will not display all the behaviours observed in whole plant tissue. However, they may prove useful for close study of initial penetrations and of haustorial activities. Conducting infections in tissue culture dishes and multiwell plates with cultured plant cells also has the potential to lend itself more readily to high‐throughput imaging platforms.

## VII. Conclusion and outlook

*Phytophthora*–host interaction research has blossomed over the last decade, particularly in the field of RXLR effector characterisation and the identification of host proteins targeted by these proteins. Effectors have proven to be powerful tools to probe the plant defence system, throwing new light on the complexity and cross‐talk between the different immune pathways, and between growth and defence. With the successful application of clustered regularly interspaced short palindromic repeats (CRISPR) for gene knockouts and other genetic manipulations in some species the stage is set for more fundamental cell biological studies on *Phytophthora* development and pathogenicity. Such studies will help to underpin research into *Phytophthora* infection biology, pathogenicity factor delivery and effector functions and, ultimately, the development of new strategies to combat these challenging pathogens.
